# Supplementary material for: Organic amendment plus inoculum drivers: Who drives more P nutrition for wheat plant fitness in small duration soil experiment
Source: PLoS One. 2022 Apr 13;17(4):e0266279. doi: 10.1371/journal.pone.0266279 (PMC9007377; doi:10.1371/journal.pone.0266279)
Supplement: S1 Table — (DOCX) [file pone.0266279.s001.docx]

| S.No | Names of bacteria | Name codes of bacteria | P mobilization (P_i_) | ALP activity |
| --- | --- | --- | --- | --- |
|  |  |  | **mg/ml** | **Moles of pNPP/ml/hr** |
| 1 | *Serratia fonticola* | Sf | 0.5668 | 0.3459 |
| 2 | *Serratia fonticola* | Sf | 0.554 | 0.332 |
| 3 | *Serratia fonticola* | Sf | 0.5469 | 0.3633 |
| 4 | *Serratia fonticola* | Sf | 0.5519 | 0.3544 |
| 5 | *Serratia fonticola* | Sf | 0.5391 | 0.3698 |
| 6 | *Serratia fonticola* | Sf | 0.5459 | 0.3763 |
| 7 | *Escherichia vulneris* | Ev | 0.4857 | 0.3687 |
| 8 | *Rhodococcus boritolerans* | Rb | 0.5797 | 0.3601 |
| 9 | *Bacillus cereus* | Bc | 0.922673 | 1.392667 |
| 10 | *Salmonella enterica* | Se | 0.3363 | 0.3831 |
| 11 | *Bacillus subtilis* | Bs | 0.981007 | 1.410333 |
| 12 | *Bacillus thuringiensis* | Bt | 1.573784 | 1.402667 |
| 13 | *Pseudomonas fluorescens* | Pf | 1.405451 | 1.420667 |
| 14 | *Pseudomonas koreensis* | Pk | 0.582 | 1.103513 |
| 15 | *Staphylococcus equorum* | Sp | 0.4986 | 0.3627 |
| 16 | *Bacillus paramycoides* | Bp | 0.485 | 0.308 |
| 17 | *Rhodococcus erythropolis* | Re | 0.4848 | 0.3991 |
| 18 | *Bacillus paramycoides* | Bp | 0.4592 | 0.3739 |
| 19 | *Glutamicibacter bergerei* | Gb | 0.5479 | 0.4205 |
| 20 | *Bacillus paramycoides* | Bp | 0.4791 | 0.397 |
| 21 | *Bacillus paramycoides* | Bp | 0.4665 | 0.3712 |

Supplementary table 1**:** ALP activity and P mobilization values by 21 bacterial strains
