# Supplementary material for: Organic amendment plus inoculum drivers: Who drives more P nutrition for wheat plant fitness in small duration soil experiment
Source: PLoS One. 2022 Apr 13;17(4):e0266279. doi: 10.1371/journal.pone.0266279 (PMC9007377; doi:10.1371/journal.pone.0266279)
Supplement: S2 Table — (DOCX) [file pone.0266279.s002.docx]

| Parameter | Factor | df | F | P |
| --- | --- | --- | --- | --- |
| Net available P  (µg/g) | Bio inoculation  Amendment  SubstrateXBio inoculation | 3  1  3 | 231.05  19.96  9.26 | 0.000  0.000  0.000 |
| ALP enzyme activity  (µ mole pNPP/ml/hr) | Bio inoculation  Amendment  SubstrateXBio inoculation | 3  1  3 | 302.752  85.845  86.758 | 0.00  0.00  0.00 |
| Plant P  (µg/g DW/pot) | Bio inoculation  Amendment  SubstrateXBio inoculation | 2  1  2 | 5.65  399.05  6.85 | 0.019  0.000  0.010 |
| Plant Dry weight  (mg/pot) | Bio inoculation  Amendment  SubstrateXBio inoculation | 2  1  2 | 1.917  47.70  0.017 | 0.190  0.000  0.983 |

Supplementary table 2:  ANOVA results showing the F and P values of significant differences between the applied treatments i.e. bioinoculation and amendment and their interaction.
