# Supplementary material for: Organic amendment plus inoculum drivers: Who drives more P nutrition for wheat plant fitness in small duration soil experiment
Source: PLoS One. 2022 Apr 13;17(4):e0266279. doi: 10.1371/journal.pone.0266279 (PMC9007377; doi:10.1371/journal.pone.0266279)

Supplementary material 4: Unadjusted and uncropped images underlying all gel figures.

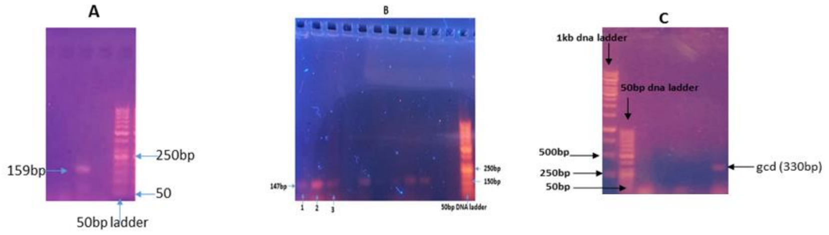

Fig A

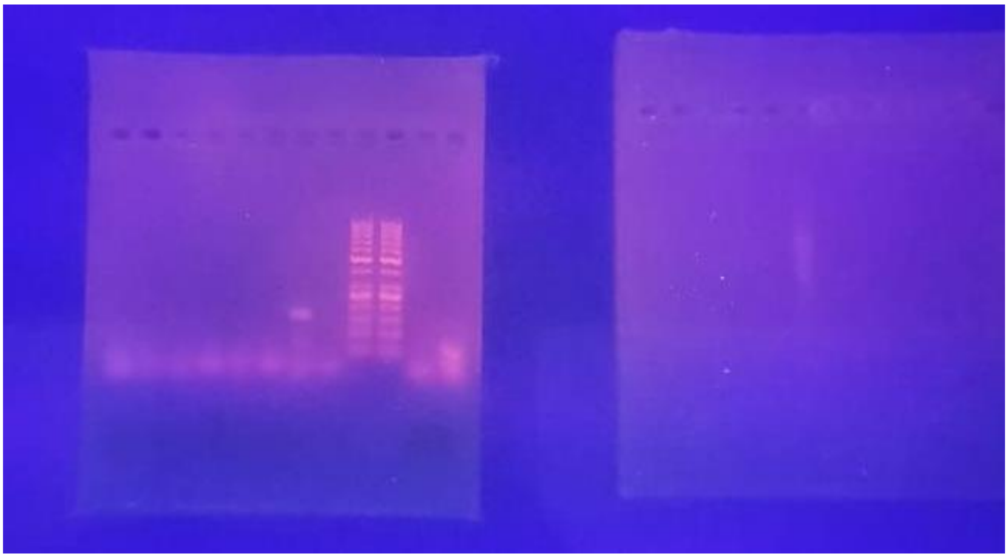

Fig B

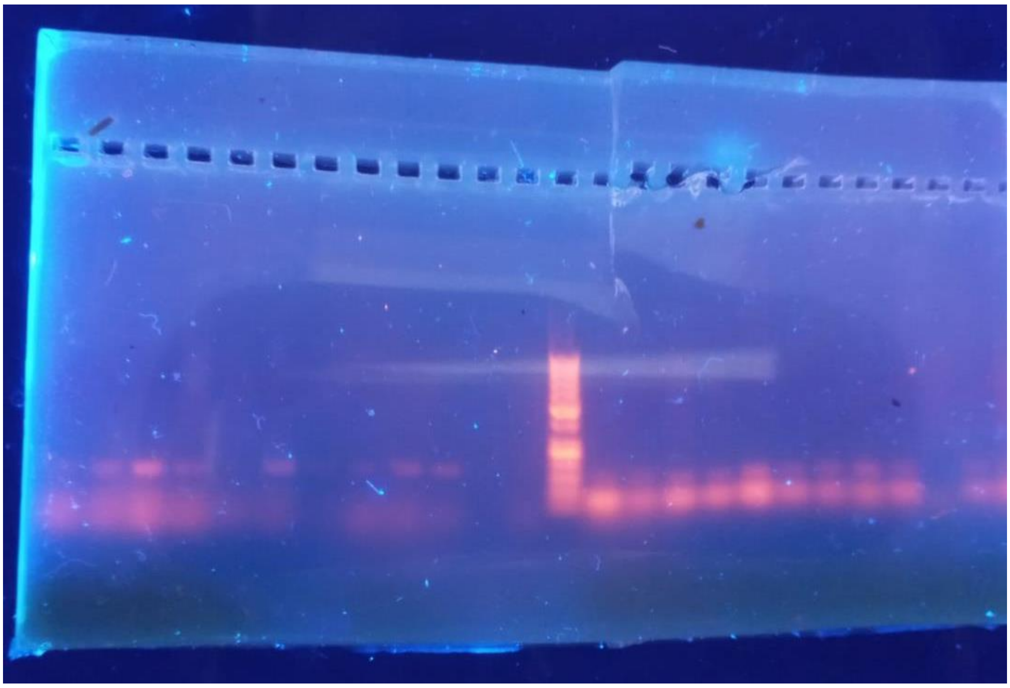

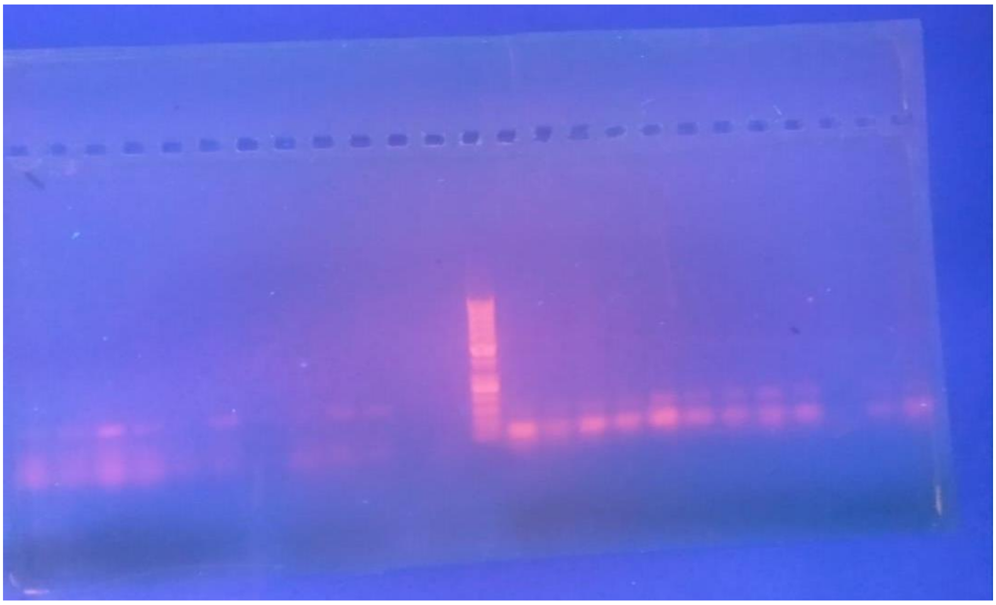

Fig c

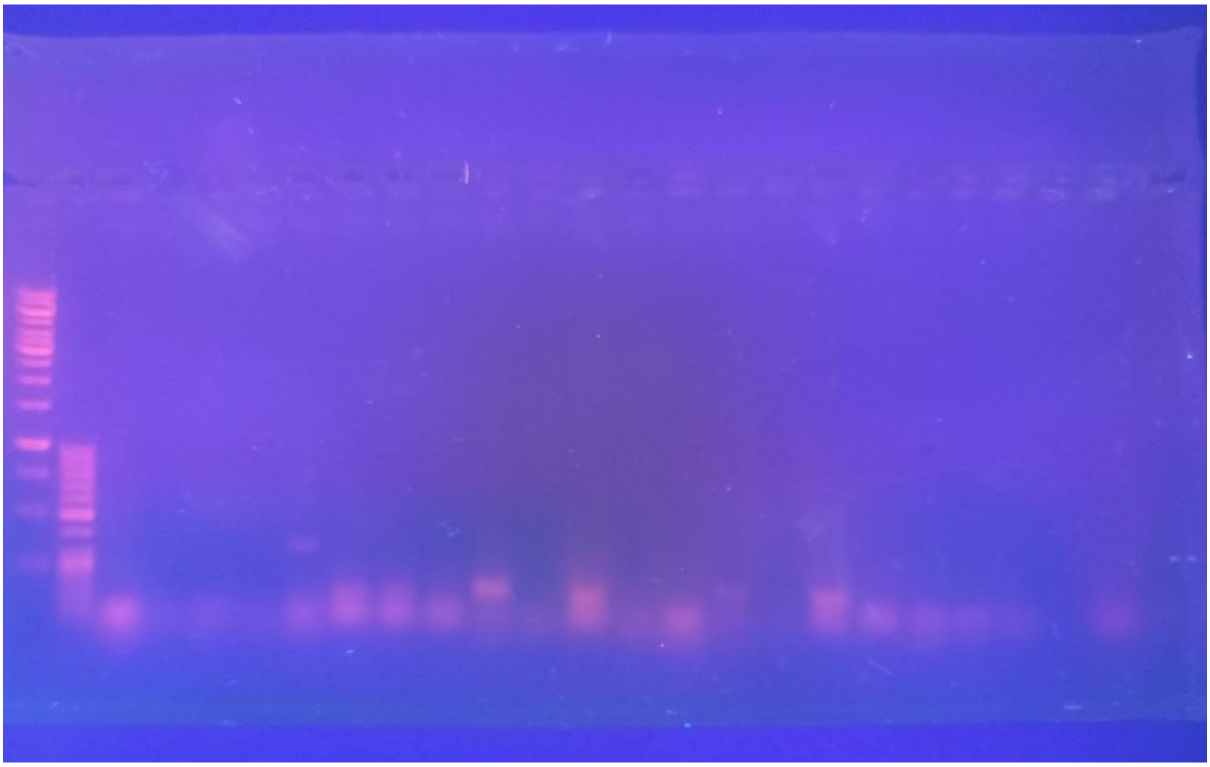

Supplement: S1 Raw images — (PDF) [file pone.0266279.s003.pdf]
