# Supplementary material for: Organic amendment plus inoculum drivers: Who drives more P nutrition for wheat plant fitness in small duration soil experiment
Source: PLoS One. 2022 Apr 13;17(4):e0266279. doi: 10.1371/journal.pone.0266279 (PMC9007377; doi:10.1371/journal.pone.0266279)
Supplement: Attachment — Submitted filename: Revision Plos ONE.docx [file pone.0266279.s005.docx]

The current study examined the potential effects of co-inoculation of phosphorus solubilizing bacteria and their grazer nematodes on soil P availability in presence of poultry manure. The subject of this article is coherent with the scope of Plos ONE. Given the depletion of the limited P-fertilizer resources, scientific understanding the interaction of soil microfauna to improve the soil P fertility is important. The manuscript is well written. However, the manuscript requires revisions before it could be accepted for publication.

**Specific comments:**

Need to define first PSB.

L122: “A synthetic solid medium” is the starting sentence of L123.

**Material and method**

A huge amount of information is presented in the mat&met section. Some sections could be presented as a supplementary information to simplify the paper.

L110-L121: The soil properties should be presented with emphasis on the soil type (P-deficient or not, alkaline or acidic soil).

L136: PKO???

L168-L169: What criteria did you choose to select the 6 efficient strains of the 21 based on pH, ALP activity and P mineralization? Please report the results even as a supplementary information.

L287-L289: Can you provide the characteristic/succinct history of the field where soil was sampled (e.g. previous crop).

L302-L3016: Summarize the tested treatments of the experimental design in table to better help readers. Specify the duration of the experiment. How did you collected the soil samples for subsequent analyses (rhizosphere or bulk for each pot).

In statistical analysis section: provide sufficient information for data handling: software, the tested treatment with the statistical approach used (here one and two-way anova). The test of normality of the data and the homogeneity of variance.

**Results:**

L376-L381: Give the Anova result either in the text or in a separate table. Describe the T1 treatment here. How did you calculate the soil net P availability in Fig 2? May be it’s better to add this information in mat&met section.

I think it’s better to present the result for each tested parameters first before going in a specific description in your text: bacteria treatment, Nematode treatment, Poultry treatment, and interactions, and should be supported by Anova results. The same also with ALP results in L388-L395, and with pH and soil net available P (L402-L406).

It is interesting to see the different response of soil net available P with soil net pH. Increasing soil pH from 5.75 to 7.5 (control to Control+wheat plant+ bacterial consortia +nematodes) increased soil P from 0.0.4 to 4 µg.g-1) in unamended treatment , while increasing soil pH from 6.5 to 7.5 increased soil P from 0.0.2 to 2.8 µg.g-1). Focusing in nematode treatment impact, how about the response of increasing soil P to soil pH between the two treatments C+P+B vs C+P+B+N with or without PM addition?

The same remarks with the net P liberation vs ALP activity.

The clear effect of Nematode under PM amendment or no organic amendment could be describe and general conclusion can be drawn from this.

I suggest that authors presents the correlation between the measured parameters as ALP, pH, soil P, and plant biomass and P uptake.

**Discussion:**

I suggest to clearly state the nematode predator effect in presence of PM and without PM on the measured parameters before discussing the underlying mechanism in L507.

L580: Fig 9???

L584: Fig 8 instead of Fig 9.

In the section “Microbial P dynamics changes in soil treatments”, L507-L555, authors stated that the predation treatment declined Net P and ALP activity with PM addition in contrast with those without PM amendment; this is related to the immobilization of available P in microbial biomass. Does it suggest that predator effect is OM-dependent in soil? Through which mechanism?

Total P uptake (P root + P shoot) could be added to clearly show the contrasting effect of treatment. Could you try to calculate the P uptake efficiency (PUPE as the ability of crops to uptake P from the soil) = P uptake/soil P available (Moll et *al.* (1982)) to verify the predator , PM treatment effects.

Do you have any results on soil available P & ALP activity measurement with time (incubation of microcosm study)? This could be interested to be related to the higher plant P uptake & biomass in PM amendment. Is it possible that after high P uptake, soil could be depleted in available P? Because here soil P & ALP seems to be negatively correlated with plant P uptake.

I consider the data from different experiments including strains screening and microcosm experiment should be highly valuable and hopefully presented. I would like to encourage the authors to present clearly the data analysis in the result section and to resubmit the revised manuscript.

I hope the specific comments may also help authors for the revision.
